# Association of Birthweight with Overweight, Obesity, and Blood Pressure among Adolescents

**DOI:** 10.3390/children10040617

**Published:** 2023-03-25

**Authors:** Hui Fan, Tao Guan, Xingyu Zhang

**Affiliations:** 1 Department of Epidemiology and Health Statistics, School of Public Health, North Sichuan Medical College, Nanchong 637100, China; 2 Department of Public Health, Liangshan Center for Disease Control and Prevention, Xichang 615000, China; 3 Thomas E.Starzl Transplantation Institute, University of Pittsburgh Medical Center, Pittsburgh, PA 15260, USA

**Keywords:** birthweight, overweight, obesity, blood pressure, adolescence

## Abstract

We investigated the association of birthweight with overweight, obesity, and blood pressure (BP) among adolescents. This cross-sectional study included 857 participants aged 11–17 years from Liangshan, southwest China. Birthweight information was collected from the participants’ parents. The participants’ height, weight, and BP were measured. High birthweight was defined as a value greater than the sex-specific upper quartile. Participants were classified into four groups based on their weight change at birth and adolescence: normal weight at both time points, weight loss, weight gain, and high weight at both time points. High birthweight was positively associated with overweight and obesity in adolescence [odds ratio (95% confidence interval), OR (95% CI): 1.93 (1.33, 2.79)]. Compared with participants with normal weight at both time points, those with high weight at both time points were more likely to have elevated BP in adolescence (OR [95% CI]: 3.02 [1.65, 5.53]), while those with weight loss had similar odds of elevated BP. The sensitivity analysis results did not change significantly when high birthweight was defined alternatively as birthweight > 4 kg. This study showed that the association between high birthweight and elevated BP in adolescence is influenced by current weight.

## 1. Introduction

Elevated blood pressure (BP) in early life is considered a major threat to public health [1] and is prevalent globally [2]. Moreover, elevated BP persists into early adulthood, contributing to the early onset of cardiovascular disease and ultimately to premature death [3,4]. Consequently, the prevention of elevated BP in early life is an effective strategy for controlling the associated disease burden [5]. The accurate identification of relevant risk factors for elevated BP in early life forms the cornerstone of prevention.

Findings on the effect of a high birthweight on elevated BP in early life are inconsistent [6,7,8,9,10,11,12,13,14,15,16,17,18]. Statistical adjustment of current weight in analyses of the association between a high birthweight and subsequent BP remain controversial [7,9,10,11,12,13,15,16,17]. Some studies have shown an independent effect of a high birthweight on BP after adjusting for current weight, given the relationship between the birthweight and current weight [7,12,15]. In contrast, other studies have considered current weight as an intermediate in the causal path from high birthweight to elevated BP later in life [9,13,16]. Statistical adjustment of current weight may introduce bias and thus ultimately generate uninterpretable results [9,13,19].

This study aimed to assess the combined influence of high birthweight and current overweight and obesity on elevated BP in adolescents. Our strategy took into account the correlation between birthweight and current weight to avoid possible bias due to over-adjustment and to facilitate the interpretation of the results. To the best of our knowledge, data are scarce regarding the combined effect of weight status from birth to adolescence on elevated BP in adolescence. Consequently, we aimed to investigate this issue in a cross-sectional study.

## 2. Material and Methods

### 2.1. Study Population

This cross-sectional study was conducted in 2021 in Liangshan, southwest China. One school from urban areas, one school from rural areas, and one school from gathering areas of Yi ethnicity were selected using convenience sampling. Each of the three selected schools included both junior and senior high schools. Three classes from grade one, grade two, and grade four were randomly selected from each school. Finally, students from 27 classes in the three schools were recruited for this study. The students were asked to complete a questionnaire and a physical examination. All of the methods were conducted in accordance with the Declaration of Helsinki. The study was approved by the Institutional Review Board of North Sichuan Medical College (approval number: 202150). Written informed consent was obtained from all of the participants and their parents or guardians. Elevated BP was considered as an indicator to obtain the required sample size. To calculate the sample size, we used the following formula: *n* = (Z_1−α/2_)^2^ × P × (1 − P)/d^2^ [20]. Using an α = 0.05, elevated BP (P) = 0.2, and a tolerance error (d)= 0.03 according to relevant publication, the required sample size was calculated to be 683 participants [21]. The study included 914 participants aged 11–17 years with complete data, which met the sample size requirement. After excluding those with a low birthweight (<2500 g, *n* = 53) or underweight in adolescence (*n* = 4), the analysis sample consisted of 857 participants [22,23].

### 2.2. Primary Exposure

Birthweight (kg) information was collected from the participants’ parents. A high birthweight was defined as a value greater than the sex-specific upper quartile for birthweight (males: 3.6 kg; females: 3.5 kg) [15]. The participants’ weight and height were measured while they wore light clothes and no shoes, using calibrated equipment to ensure the accuracy of the measurements. The body mass index (BMI) was calculated as the weight divided by the square of the height (kg/m^2^). Overweight and obesity in adolescence were defined as a BMI greater than or equal to the corresponding sex- and age-specific overweight cutoffs using the overweight and obesity screening criteria for Chinese children and adolescents [24]. The participants were classified into four groups based on the birthweight and weight in adolescence: normal weight at both time points (birth and adolescence), weight loss (high birthweight and normal weight in adolescence), weight gain (normal birthweight and overweight and obesity in adolescence), and high weight at both time points (high birthweight and overweight and obesity in adolescence).

### 2.3. Outcome Assessment

After a rest period of at least 10 min, the participants’ BP was measured using an electronic sphygmomanometer (HBP-1320; Omron Healthcare, China) that had previously passed validation protocols [25]. Trained physicians asked the participants to assume a sitting position and chose cuffs that were appropriately sized for the participants’ right arms. BP was measured three times, and two consecutive measurements were separated by at least a 30-s interval. The mean of the final two BP measurements was used for further analyses. Elevated BP was defined as a BP measurement greater than or equal to the corresponding sex-, age-, and height-specific 90th percentile value, 120/80 mm Hg, or the use of antihypertensive drugs [26].

### 2.4. Covariates

Self-reported data on the participants’ sex (male/female), ethnicity (Han/Yi/other), school, age, sleep duration, physical activity, and intake of whole grains were collected. Physical inactivity was defined as less than 60 min of moderate–vigorous physical activity per day [27]. Insufficient intake of whole grains was defined as less than once per day. Data on preterm birth, breastfeeding, and family history of hypertension were collected from the participants’ parents. Preterm birth and breastfeeding were defined as fewerthan 37 complete weeks of gestation and breastfeeding for 6 months after birth, respectively [28,29]. A family history of hypertension was defined as having at least one parent who had ever been diagnosed with hypertension.

### 2.5. Statistical Analysis

Means ± standard deviations and numbers (percentages) are used to describe continuous and categorical variables, respectively. A student’s t test and a chi-square test were used to compare continuous and categorical variables, respectively, between participants with normal and high birthweights.

After adjustment for sex, ethnicity, school, preterm birth, breastfeeding, age, sleep duration, physical inactivity, and insufficient intake of whole grains, linear and logistic regression models were used to investigate the associations of birthweight with BMI and overweight and obesity in adolescence, respectively.

We used linear and logistic regression models to evaluate the independent effects of birthweight on systolic BP (a continuous variable), diastolic BP (a continuous variable), and elevated BP (a categorical variable) in adolescence. We conducted similar analyses to assess the independent effects of current overweight and obesity on systolic BP, diastolic BP, and elevated BP in adolescence. We also analyzed the associations between birthweight, current overweight and obesity, and systolic BP, diastolic BP, and elevated BP in adolescence by simultaneously including birthweight and current overweight and obesity in the models.

We also used linear and logistic regression models to assess the combined influence of birthweight and overweight and obesity in adolescence on systolic BP, diastolic BP, and elevated BP in adolescence. To analyze the trend in the prevalence of elevated BP across weight changes from birth to adolescence, we considered the weight change from birth to adolescence as a continuous variable and assigned respective values of 0, 1, 2, and 3 to the following group assignments: normal weight at both time points, weight loss, weight gain, and high weight at both time points. We then used logistic regression models to analyze these data.

To test the robustness of our findings, we repeated all of the analyses after defining high birthweight alternatively as a birthweight > 4 kg [30].

We used SAS 9.4 (SAS Institute, Cary, NC, USA) to perform all of the statistical analyses. We considered a two-sided *p* value < 0.05 to indicate statistical significance.

## 3. Results

Table 1 summarizes the characteristics of the participants stratified by birthweight. The normal and high birthweight groups consisted of 662 participants (males: 42.6%; mean age: 13.9 years) and 195 participants (males: 42.1%; mean age: 13.7 years), respectively.

Table 2 presents an evaluation of birthweight in relation to weight status in adolescence. Participants with a high birthweight had a higher BMI in adolescence than those with a normal birthweight (β [SE]: 1.28 [0.27]; *p* < 0.001]. Overweight and obesity in adolescence was observed in 31.3% and 19.2% of the participants with a high and normal birthweight, respectively. Compared with participants with a normal birthweight, those with a high birthweight were more likely to experience overweight or obesity in adolescence [odds ratio, OR (95% confidence interval, CI): 1.93 (1.33, 2.79)].

Table 3 presents the independent effects of birthweight and current overweight and obesity on elevated BP in adolescence. The independent effects of high birthweight on systolic BP, diastolic BP, and elevated BP in adolescence were not statistically significant after adjusting for covariates (Model 1; all *p* > 0.05). However, current overweight and obesity had significant independent effects on systolic BP, diastolic BP, and elevated BP in adolescence (Model 2; all *p* < 0.05). When the models simultaneously included birthweight and current overweight and obesity, the associations of current overweight and obesity with systolic BP, diastolic BP, and elevated BP remained significant (Model 3; all *p* < 0.05).

Table 4 presents the association between the weight change from birth to adolescence and elevated BP in adolescence. Compared to adolescents with a normal weight at both birth and adolescence, those with a high weight at both time points had a higher systolic BP (β [SE]: 6.76 (1.47); *p* < 0.001) and were more likely to have elevated BP in adolescence (OR [95% CI]: 3.02 [1.65, 5.53]). Those with weight gain had a higher systolic BP (β [SE]: 8.14 [1.08]; *p* < 0.001) and were more likely to have elevated BP in adolescence (OR [95% CI]: 3.77 [2.39, 5.94]) than those with a normal weight at both time points, whereas those with weight loss did not have increased odds of elevated BP in adolescence (OR [95% CI]: 1.55 [0.94, 2.56]). The prevalence of elevated BP was 15%, 20.2%, 38.6%, and 34.4% in participants with a normal weight at both time points, those with weight loss, those with weight gain, and those with a high weight at both time points, respectively. A significant increasing trend in the prevalence of elevated BP was observed across the four groups (*p* < 0.001).

We repeated all of the analyses using the alternate definition of a high birthweight (birthweight > 4 kg). The results of the sensitivity analysis did not differ significantly from the initial results (Supplementary Tables S1–S3).

## 4. Discussion

This study found that a high birthweight was positively associated with overweight and obesity in adolescence. Compared with participants with a normal weight at both birth and adolescence, those with a high weight at both time points and those with weight gain were more likely to have elevated BP in adolescence, whereas those with weight loss had similar odds of elevated BP in adolescence.

Overweight and obesity in adolescence were observed in 31.3% of the participants with a high birthweight, indicating a persistent trend in high weight from birth to adolescence. We also found that participants with a high birthweight had 1.93-times higher odds of experiencing overweight and obesity in adolescence, consistent with the findings of cross-sectional and cohort studies [31,32,33,34]. A meta-analysis also found that a high birthweight is a determinant of overweight and obesity later in life [35]. Several potential factors, including genetic, fetal nutrition, and lifestyle factors, were used to explain these findings [32].

This study found no independent effect of a high birthweight on elevated BP in adolescence, regardless of adjustment for current overweight and obesity, which is consistent with the findings of some studies [10,14]. However, other studies have reported significantly inverse or positive independent effects of birthweight on subsequent BP levels and elevated BP [6,7,11,15,16,17,18]. These discrepancies are partly attributable to differences between studies’ design, inclusion/exclusion criteria, methods used to access information on birthweight, time points at which BP was measured, and birthweight distributions in studies’ populations. Moreover, statistical adjustment of current weight may introduce bias and obfuscate findings, resulting in inconsistencies [9,13,19]. Consequently, to avoid the abovementioned problems, analysis of the combined influence of birthweight and current weight on elevated BP is recommended.

In this study, compared with participants with a normal weight at both birth and adolescence, those with a high weight at both time points and those with weight gain were more likely to have elevated BP in adolescence, while those with weight loss had similar odds of elevated BP in adolescence. Our findings highlight the influence of current weight on the relationship between birthweight and elevated BP in adolescence. A cross-sectional study of preschoolers in southeast China used a similar strategy and found that elevated BP was determined by current weight, regardless of birthweight [16]. The results of another study partly support our findings, as the authors showed that children with excess weight both at birth and in childhood and those with weight gain were more likely to have a high carotid intimal–medial thickness in childhood than children with a normal weight at both time points [30]. An increased carotid intimal–medial thickness is indicative of hypertensive target organ damage.

Liangshan is a developing region in the Sichuan province, southwest China. Our findings show there is a high prevalence of overweight and obesity and elevated BP in adolescents in Liangshan, which is similar to China’s national epidemic level [21,36]. This indicates that attention should be paid to the epidemic of overweight and obesity and associated elevated BP in early life, even in developing regions of China. Weight control during pregnancy, exclusive breastfeeding, intake of a healthy diet, multi-sectoral coordination, and involvement of all aspects of society may play important roles in addressing the abovementioned epidemic [37,38,39].

Our study has some strengths, including the use of professional investigators, validated equipment, standard data-collection procedures, and statistical adjustment of covariates. However, several limitations should be acknowledged. First, birthweight data were mainly dependent on parental recall, which may have introduced recall bias. However, mothers’ recall of their children’s birthweight is a valid alternative to medical records [40]. Additionally, the parents of adolescents who were born in hospitals may have recorded documentation of their children’s birthweight. Second, elevated BP in adolescence was identified using measurements taken at a single time point, which may have led to overestimation of this condition due to the high variability in BP in adolescence [1]. Third, convenience sampling was used to select the included schools, which may have affected the generalizability of our results. Fourth, we were unable to assess observer variation due to limitations in our study design. However, we recruited well-trained medical staff with relevant clinical work experience, which may have reduced any associated bias. Fifth, although Liangshan is a multi-ethnic area, the sample size was insufficient to conduct sensitivity analyses stratified by ethnicity. Moreover, the sample size may have been insufficient to conduct subgroup analyses stratified by preterm birth status. Finally, a causal relationship between the weight change from birth to adolescence and elevated BP in adolescence could not be determined in this cross-sectional study. A cohort study with a large sample size will be needed to confirm our results.

## 5. Conclusions

In summary, this study showed there is a significant association between a high birthweight and overweight and obesity in adolescence. Our study further demonstrated that participants with a high weight at both birth and adolescence and those with weight gain were more likely to have elevated BP than those with a normal weight at both time points, whereas those with weight loss did not have increased odds of elevated BP compared with the normal weight group. These results show that the association between high birthweight and elevated BP in adolescence is influenced by body weight in adolescence.

## Figures and Tables

**Table 1:** Characteristics of the participants stratified by birthweight.

	Normal Birthweight (*N* = 662)	High Birthweight (*N* = 195)	*p*
Demographic information			
Male, *n* (%)	282 (42.6%)	82 (42.1%)	0.892
Ethnicity, *n* (%)			0.233
Han	303 (45.8%)	77 (39.5%)	
Yi	338 (51.1%)	109 (55.9%)	
Others	21 (3.2%)	9 (4.6%)	
School, *n* (%)			0.818
A	250 (37.8%)	69 (35.4%)	
B	238 (36.0%)	74 (38.0%)	
C	174 (26.3%)	52 (26.7%)	
At birth			
Birthweight, kg	3.1 ± 0.3	4.1 ± 3.2	<0.001
Preterm birth, *n* (%)	31 (4.7%)	7 (3.6%)	0.515
Breastfeeding, *n* (%)	592 (89.4%)	177 (90.8%)	0.587
In adolescence			
Age, years	13.9 ± 1.4	13.7 ± 1.4	0.256
Sleep duration, hours	8.4 ± 0.8	8.4 ± 0.8	0.722
Physical inactivity, *n* (%)	392 (59.2%)	118 (60.5%)	0.746
Insufficient intake of whole grain, *n* (%)	375 (56.7%)	110 (56.4%)	0.953
Family history of hypertension, *n* (%)	57 (8.6%)	18 (9.2%)	0.788
SBP, mm Hg	106.9 ± 11.7	108.3 ± 12.2	0.124
DBP, mm Hg	61.9 ± 9.1	62.1 ± 9.0	0.824
Elevated BP, *n* (%)	129 (19.5%)	48 (24.6%)	0.120

BP, blood pressure; DBP, diastolic blood pressure; SBP, systolic blood pressure. Mean ± SD and number (percentage) were used to describe the continuous and categorical variables, respectively.

**Table 2:** Birthweight in relation to weight status in adolescence.

	Normal Birthweight (*N* = 662)	High Birthweight (*N* = 195)
BMI in adolescence, kg/m^2^		
Mean ± SD	20.3 ± 3.2	21.5 ± 3.8
β (SE) *	ref	1.28 (0.27)
*p*		<0.001
Overweight and obesity in adolescence		
Prevalence, %	19.2	31.3
OR (95%CI) *	ref	1.93 (1.33, 2.79)
*p*		<0.001

BMI, body mass index; OR, odds ratio; CI, confidence interval. * Adjusting for sex, ethnicity, school, preterm birth, breastfeeding, age, sleep duration, physical inactivity, and insufficient intake of whole grain.

**Table 3:** Independent effects of birthweight and current overweight and obesity on elevated BP in adolescence.

	SBP		DBP		Elevated BP	
	β (SE) *	*p*	β (SE) *	*p*	OR (95%CI) *	*p*
Model 1						
High birthweight	1.71 (0.91)	0.061	0.20 (0.74)	0.783	1.41 (0.95, 2.08)	0.087
Model 2						
Overweight and obesity	7.34 (0.91)	<0.001	1.89 (0.76)	0.013	3.19 (2.17, 4.68)	<0.001
Model 3						
High birthweight	0.87 (0.89)	0.326	−0.02 (0.74)	0.981	1.21 (0.81, 1.82)	0.351
Overweight and obesity	7.23 (0.92)	<0.001	1.89 (0.77)	0.014	3.11 (2.11, 4.58)	<0.001

BP, blood pressure; DBP, diastolic blood pressure; SBP, systolic blood pressure; OR, odds ratio; CI, confidence interval. * Adjusting for sex, ethnicity, school, preterm birth, breastfeeding, age, sleep duration, physical inactivity, insufficient intake of whole grain, and family history of hypertension.

**Table 4:** Association of weight change from birth to adolescence with elevated BP in adolescence.

	Normal Weight at Both Time Points (*n* =535)	Weight Loss (*n* =134)	Weight Gain (*n* =127)	Excess Weight at Both Time Points (*n* =61)
SBP, mm Hg				
Mean ± SD	105.4 ± 10.6	106.6 ± 11.6	113.1 ± 13.9	112.2 ± 12.7
β (SE) *	ref	1.75 (1.05)	8.14 (1.08)	6.76 (1.47)
*p*		0.096	<0.001	<0.001
*p* for trend	<0.001			
DBP, mm Hg				
Mean ± SD	61.5 ± 8.5	61.4 ± 7.7	63.8 ± 11.2	63.5 ± 11.4
β (SE) *	ref	0.13 (0.87)	2.04 (0.91)	1.66 (1.23)
*p*		0.886	0.024	0.178
*p* for trend	0.026			
Elevated BP				
Prevalence, %	15.0	20.2	38.6	34.4
OR (95%CI) *	ref	1.55 (0.94, 2.56)	3.77 (2.39, 5.94)	3.02 (1.65, 5.53)
*p*		0.084	<0.001	<0.001
*p* for trend	<0.001			

BP, blood pressure; DBP, diastolic blood pressure; SBP, systolic blood pressure; OR, odds ratio; CI, confidence interval. * Adjusting for sex, ethnicity, school, preterm birth, breastfeeding, age, sleep duration, physical inactivity, insufficient intake of whole grain, and family history of hypertension.

## Data Availability

The datasets generated or analyzed during the current study are available from the corresponding author upon reasonable request.

## Supplementary Materials

The following supporting information can be downloaded at: https://www.mdpi.com/article/10.3390/children10040617/s1, Table S1: Birthweight in relation to weight status in adolescence; Table S2: Independent effects of birthweight and current overweight and obesity on elevated BP in adolescence; Table S3: Association of weight change from birth to adolescence with elevated BP in adolescence.
