# Supplementary material for: Association of Birthweight with Overweight, Obesity, and Blood Pressure among Adolescents
Source: Children (Basel). 2023 Mar 25;10(4):617. doi: 10.3390/children10040617 (PMC10136756; doi:10.3390/children10040617)
Supplement: Supplementary file 1 [file children-10-00617-s001.zip › children-2282323-supplementary.pdf]

Table S1. Birthweight in relation to weight status in adolescence

	Normal birthweight (N=809)	High birthweight (N=48)
BMI in adolescence, kg/m^2		
Mean±SD	20.5±3.4	21.4±3.9
β (SE)*	ref	0.99 (0.49)
P		0.046
Overweight and obesity in adolescence		
Prevalence, %	21.1	35.4
OR (95%CI) *	ref	2.04 (1.08, 3.86)
P		0.029

BMI, body mass index; OR, odds ratio; CI, confidence interval.

* Adjusting for sex, ethnicity, school, preterm birth, breastfeeding, age, sleep duration, physical inactivity, and insufficient intake of whole grain.

Table S2. Independent effects of birthweight and current overweight and obesity on elevated BP in adolescence

	OR (95%CI) *	*P*
Model 1		
High birthweight	1.36 (0.69, 2.69)	0.379
Model 2		
Overweight and obesity	3.19 (2.17, 4.68)	<0.001
Model 3		
High birthweight	1.15 (0.57, 2.33)	0.700
Overweight and obesity	3.17 (2.15, 4.65)	<0.001

BP, blood pressure; OR, odds ratio; CI, confidence interval.

* Adjusting for sex, ethnicity, school, preterm birth, breastfeeding, age, sleep duration, physical inactivity, insufficient intake of whole grain, and family history of hypertension

Table S3. Association of weight change from birth to adolescence with elevated BP in adolescence

	Normal weight at both time points (n=638)	Weight loss (n=31)	Weight gain (n=171)	Excess weight at both time points (n=17)
SBP, mm Hg				
Mean±SD	105.4±10.8	109.8±11.4	112.7±13.1	113.4±17.2
β (SE)*	ref	4.04 (1.99)	7.59 (0.95)	7.11 (2.66)
P		0.043	<0.001	0.008
P for trend		<0.001		
DBP, mm Hg				
Mean±SD	61.4±8.4	63.0±6.3	63.6±11.0	65.1±13.5
β (SE)*	ref	1.45 (1.66)	1.80 (0.79)	3.59 (2.22)
P		0.385	0.024	0.107
P for trend		0.007		
Elevated BP				
Prevalence, %	15.8	19.4	36.8	41.2
OR (95%CI) *	ref	1.18 (0.46, 3.04)	3.18 (2.13, 4.75)	3.52 (1.26, 9.88)
P		0.726	<0.001	0.017
P for trend		<0.001		

BP, blood pressure; DBP, diastolic blood pressure; SBP, systolic blood pressure; OR, odds ratio; CI, confidence interval.

* Adjusting for sex, ethnicity, school, preterm birth, breastfeeding, age, sleep duration, physical inactivity, insufficient intake of whole grain, and family history of hypertension.
